# Are Ocular Markers of Sun Exposure Associated With Skin Cancer? A Cross‐Sectional Analysis of the Busselton Healthy Ageing Study

**DOI:** 10.1111/ceo.70148

**Published:** 2026-07-27

**Authors:** Gareth Lingham, Vinay Kumar Nilagiri, Holly Brown, Chrianna Bharat, Samantha S. Y. Lee, Maria Franchina, Michael Hunter, Kevin Murray, David Alonso‐Caneiro, Catherine M. Olsen, David C. Whiteman, David A. Mackey

**Affiliations:** ^1^ Centre for Ophthalmology and Visual Science (Incorporating Lions Eye Institute) University of Western Australia Perth Australia; ^2^ Centre for Eye Research Ireland, Environment, Sustainability and Health Research Hub Technological University Dublin Dublin Ireland; ^3^ Centre for Eye Research Australia Melbourne Australia; ^4^ School of Population and Global Health University of Western Australia Perth Australia; ^5^ National Drug and Alcohol Research Centre University of New South Wales Sydney Australia; ^6^ School of Optometry and Vision Science University of New South Wales Sydney Australia; ^7^ Busselton Population Medical Research Institute Busselton Australia; ^8^ School of Science, Technology, and Engineering University of the Sunshine Coast Petrie Australia; ^9^ Contact Lens and Visual Optics Laboratory, Centre for Vision and Eye Research, Optometry and Vision Science Queensland University of Technology Kelvin Grove Australia; ^10^ Cancer Control Group QIMR Berghofer Medical Research Institute Brisbane Australia; ^11^ Dermatology Research Centre, Frazer Institute The University of Queensland Brisbane Australia; ^12^ Faculty of Medicine The University of Queensland Brisbane Australia

**Keywords:** Australia, eye, myopia, pterygium, skin cancer

## Abstract

**Background:**

Higher past sun or ultraviolet radiation exposure increases the risk of skin cancer and three common ocular findings: pterygium, conjunctival ultraviolet autofluorescence (CUVAF) and myopia. We investigated whether ocular markers of past sun exposure are associated with a history of skin cancer.

**Methods:**

A cross‐sectional study of participants who attended the Phase 2 examination of the Busselton Healthy Ageing Study in Western Australia (2016–2022). Myopia was determined from autorefraction and pterygium and CUVAF from photographs of the anterior eye. Past diagnosis of a skin cancer was obtained from linked hospitalisation and cancer registry data (melanoma) or combining linked and self‐reported data (keratinocyte cancer). Multivariable logistic regression with multiple imputation was used for analysis.

**Results:**

Of 3888 participants who attended, 3829 (98.5%) had skin cancer history data (mean [standard deviation] age 64.2 [5.4] years). A history of keratinocyte cancer was present in 795 (20.8%) participants and cutaneous melanoma in 59 (1.5%) participants. After adjustments, keratinocyte skin cancer was associated with a current pterygium (odds ratio [OR] = 1.33, 95% confidence interval [CI]: 1.05, 1.69) and a previous self‐reported pterygium excision (OR = 1.85, 95% CI: 1.21, 2.82). Neither CUVAF or myopia was associated with keratinocyte cancer or melanoma diagnoses.

**Conclusions:**

Having a current pterygium or previous pterygium excision is associated with a diagnosis of keratinocyte skin cancer. While causality cannot be determined, the association may reflect shared risk factors such as chronic ultraviolet radiation exposure, and people with a pterygium should be advised that they are at higher risk of skin cancer.

## 1 Introduction

Australia and New Zealand have the highest global incidences of cutaneous melanoma [1], and melanoma is a top‐10 leading cause of death from cancer in Australia [2]. While associated with lower mortality, keratinocyte skin cancers—comprising the two most common non‐melanoma skin cancers, basal cell carcinoma (BCC) and squamous cell carcinoma (SCC)—place a high burden on Australian healthcare systems [3]. In 2023–2024, the 12‐month healthcare cost of cutaneous melanoma and keratinocyte cancer in Australia was approximately AUD $2.5 billion [4].

Accurately stratifying Australians into high and low risk groups for developing skin cancers would enable increased individual awareness, monitoring (if cost‐effective) and early intervention, potentially reducing the burden of skin cancers. For example, the mean per‐patient first‐year cost of melanoma treatment is far cheaper if detected early (AUD $644 for melanoma in situ) compared to later (AUD $100 725 for unresectable, advanced melanoma) [5]. Exposure to ultraviolet radiation (UVR; 100–400 nm) is the major, modifiable risk factor for both cutaneous melanoma and keratinocyte cancers. Assessment of past solar UVR exposure—alongside non‐modifiable risk factors of older age, fairer skin colour, a high number of melanocytic naevi and immunodeficiency [6, 7, 8]—could improve the precision of skin cancer risk stratification. However, accurate assessment of past solar UVR exposure remains a challenge with subjective responses exposed to recall bias and often overestimating actual exposure [9, 10, 11]. Subjective responses become even less reliable when recalling solar UVR in the distant past (> 10 years prior) [12]. Objective biomarkers, that typically represent cumulative UVR exposure over months and years [13], are valuable alternatives to subjective recall and may more accurately stratify individuals into high and low past UVR exposure.

Alongside the skin, the eye is uniquely exposed to solar UVR, which may manifest in the development of clinical ophthalmic helioses such as pterygium, cataract or cancers of the eye (Figure 1) [14]. Sub‐clinically, exposure to solar UVR may also manifest as conjunctival ultraviolet autofluorescence (CUVAF) [15], with a larger CUVAF area correlated with greater past sun [16] and ambient UVR exposure [17]. Conversely, myopia (short‐sightedness) is linked to low amounts of time spent outdoors during childhood and adolescence [13, 18] and is associated with lower CUVAF area [19], making it a marker of low solar UVR exposure in early life. Myopia, CUVAF and pterygium are all common, easily assessed, objective ocular markers that reflect past exposure to UVR. This study aimed to assess whether having a pterygium, a larger CUVAF area or not having myopia is associated with a higher prevalence of skin cancer in a cross‐sectional analysis of the Busselton Healthy Ageing Study (BHAS) cohort.

**FIGURE 1:**
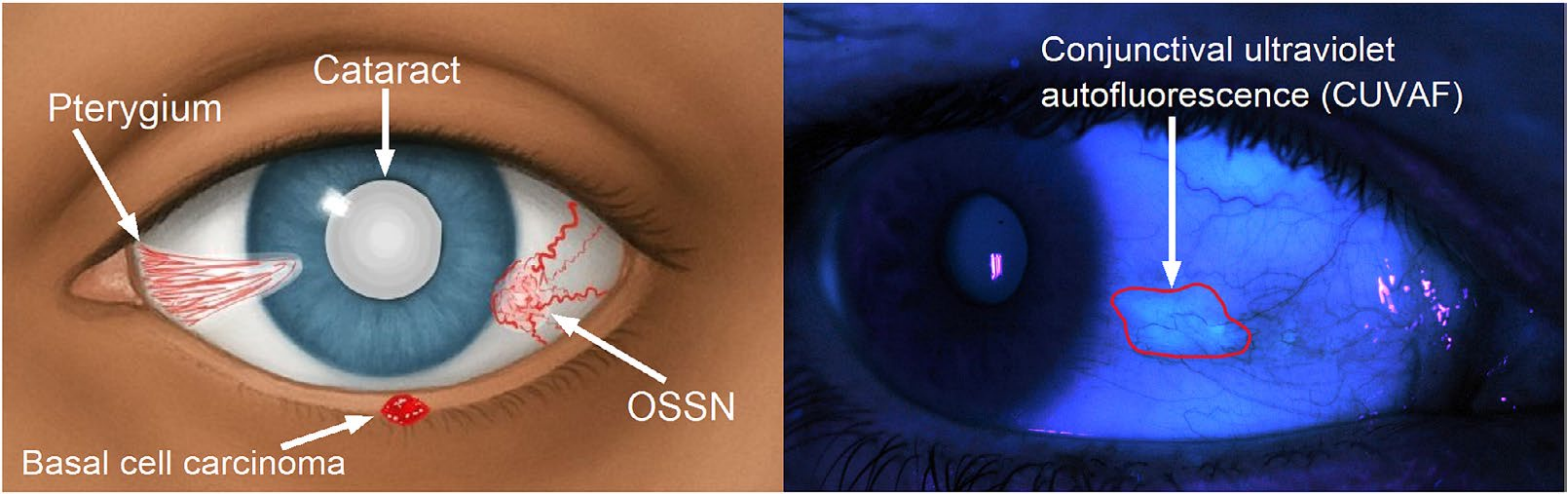
Examples of the visible effects of ultraviolet radiation exposure on the eye: (left) illustration showing a pterygium, cataract, ocular surface squamous neoplasia (OSSN) and a basal cell carcinoma of the eye lid (a skin cancer) and (right) example of a mild to moderate amount of conjunctival ultraviolet autofluorescence (CUVAF; approximately outlined in red) on the nasal conjunctiva.

## 2 Methods

The population‐based BHAS cohort was established as a study of ageing and multimorbidity under the long‐running Busselton Health Study [20]. Between 2010 and 2015, all non‐institutionalised adults born between 1946 and 1964 and listed on the electoral roll as residing in the shire of Busselton (33.7° South), Western Australia (WA), were invited to attend a baseline examination (Phase 1). Of those invited, 82% were contactable and 76% (*n* = 5107) participated. Between 2016 and 2022, participants were invited to attend a follow‐up visit (Phase 2).

Participants were included in the current analysis if they had information on history of skin cancer diagnosis. The analysis was restricted to participants who self‐reported a European ancestry/ethnicity, as a proxy for populations with fairer skin and consequently higher risk of skin cancer. Other ancestral subpopulations were not analysed due to small sample size (1% of total sample). We also excluded participants using any of the following immunosuppressant medications that are known to increase skin cancer risk: adalimumab, etanercept, infliximab, azathioprine, methotrexate and tacrolimus [7, 8].

### 2.1 Exposure Assessments

Pterygium and CUVAF area were assessed as previously described [21]. Briefly, pterygium was assessed by a single grader (H.B.) from monocular colour photographs of the anterior eye captured in primary position and looking left and right (D250, Nikon, Japan). Photographs where the presence/absence of a pterygium was unclear were adjudicated by an ophthalmologist (M.F. and D.A.M.). A participant with pterygium in either or both eyes was classified as having pterygium [16]. Participants self‐reported any past eye surgeries or procedures at Phase 2, from which we categorised previous pterygium excision. Iris colour was graded from colour photographs following a published schema [22] and grouped into blue (Grades 1–3), green (Grades 4–5), hazel (mix of green and brown; Grades 6–7) and brown (Grades 8–9).

CUVAF photographs of the nasal and temporal conjunctiva of both eyes were captured using our previously described setup [21]. CUVAF area was measured using a supervised deep‐learning tool [21] by a single grader (H.B.). Because some participants had images from only one eye, the mean CUVAF area of the four (or two) quadrants was used as the exposure variable.

Myopia was classified from autorefraction at Phase 1, after administration of 1% tropicamide eye drops (ARK‐30, Nidek, Japan) [23]. Phase 1 was used instead of Phase 2 because we aimed to minimise the number of participants excluded due to self‐reported cataract or having had cataract surgery, both of which can change refraction [23]. Due to a device malfunction, measurements taken between January 2013 and July 2014, inclusive, were discarded (approximately 30%), reducing the sample size for analyses involving myopia [23]. Myopia was defined as a spherical equivalent refractive error equal to or more myopic than −0.50 D in both eyes, or in one eye for monocular participants.

### 2.2 Outcome Assessment

At both visits, participants were asked to self‐report past diagnoses of cancer by a doctor and to specify the type of cancer and age at diagnosis in free text. Data from both Phases 1 and 2 were used to classify self‐reported skin cancer history.

Linked (administratively recorded) data from the WA Department of Health also provided records of both melanoma and keratinocyte cancer diagnoses with the WA Cancer Registry, hospitalisations and death data. Since 1982, melanoma, both invasive and in situ, has been a compulsory notifiable condition in WA, while keratinocyte cancer diagnoses may be optionally registered. For hospitalisations and death data, which extends back to 1980, melanoma skin cancer was classified from International Classification of Diseases (ICD)‐9 code 172 and ICD‐10 codes C43 and D03, while keratinocyte skin cancer was classified from ICD‐9 codes 173 and 232 and ICD‐10 codes C44 and D04. These ICD codes cover both in situ and invasive skin cancers. ICD‐9 code 232 includes both in situ carcinomas and melanomas without differentiating between the two. We recorded these as keratinocyte cancers as the more likely response. Melanomas of the genitalia were not captured as these would typically be unrelated to UV radiation.

Because cutaneous melanoma is a compulsory notifiable condition, diagnoses from administrative records should capture all cases diagnosed since 1982 when the WA cancer registry was established. Thus, a positive melanoma history was recorded if a participant had either:

- an administrative record of melanoma or
- a self‐reported diagnosis of melanoma with a year of diagnosis prior to 1982.

Because registration of keratinocyte cancer is optional, administrative data will underestimate the true number of cases; therefore, a mixture of administrative and self‐reported data was used to determine keratinocyte cancer history. A positive keratinocyte cancer diagnosis was recorded if a participant had either:

- an administrative record of keratinocyte cancer,
- a self‐reported diagnosis of BCC or SCC or
- a self‐reported diagnosis of a non‐specific skin cancer, or melanoma skin cancer, without meeting the classification criteria for melanoma. This presumes that melanoma was mis‐reported if no administrative record is present.

Age at cancer diagnosis was self‐reported by participants and we used the youngest reported age where more than one was available [13, 24, 25].

### 2.3 Statistical Analysis

Participants were excluded from the analysis if they were missing outcome data (keratinocyte or melanoma diagnosis). Agreement between self‐reported and administratively captured skin cancer history was evaluated using the *κ* coefficient and interpreted using published thresholds (0: poor, 0–0.2: slight, 0.21–0.4: fair, 0.41–0.6: moderate, 0.61–0.8: substantial, 0.81–1: almost perfect) [24]. Participants with self‐reported age of onset of melanoma or keratinocyte cancer prior to 1982 or 1980, respectively, were excluded. Skin cancer—either melanoma or keratinocyte cancer—was treated as separate binary outcomes and analysed using logistic regression. Covariates included age, sex, eye colour (as a proxy for skin colour), self‐reported family history of skin cancer in first‐degree relatives and self‐reported smoking history (never vs. ever) [6, 25, 26]. To mitigate the impact of missing exposure and covariate data on associations, we used multiple imputation with chained equations. Missing exposure data were assumed to be missing at random as most missing data were a result of equipment malfunction, thus related to visit date. Input data for imputations included all outcomes, exposures and covariates as well as questionnaire variables on sun tanning, self‐reported time outdoors and sun protection behaviours. Logistic regression and predictive mean matching were used to impute binary and continuous variables, respectively, and 40 imputed datasets were generated with 20 iterations. As the key exposures were only assessed at either Phase 1 or Phase 2, the present analysis is cross‐sectional and we adopted a ‘causal’ inference strategy, evaluating the strength and size of associations without being able to determine directionality. To minimise the impact of ambient UV environments during early life, a sensitivity analysis limited to participants born in Australia was conducted. Analyses were conducted using R version 4.3.2 (R Foundation for Statistical Computing, Vienna, Austria).

### 2.4 Ethics Approval

The BHAS follow‐ups were approved by the University of Western Australia Human Research Ethics Committee (RA/4/1/2203, 2021/ET000260) and participants provided written informed consent prior to participating.

## 3 Results

### 3.1 Characteristics

Of 5107 participants who attended the Phase 1 BHAS examination, 3888 (76.2%) returned for Phase 2. After excluding participants with non‐European ancestry (*n* = 40) or taking an immunosuppressive medication (*n* = 17), 3829 (98.5%) participants were included. Participants' characteristics are shown in Table 1. A positive skin cancer history was present in 803 (21.0%) participants, of whom 744 (92.7%) had only a keratinocyte cancer diagnosis, 8 (1.0%) had only a cutaneous melanoma diagnosis and 51 (6.4%) had both.

**TABLE 1:** Characteristics of study participants by self‐reported skin cancer history.

Characteristics	No skin cancer history	Skin cancer history	Overall
*N*	3026 (79.0%)	803 (21.0%)	3829
Age (years)	63.8 (5.4)	65.5 (5.3)	64.2 (5.4)
Sex			
Female	1706.0 (56.4%)	402.0 (50.1%)	2108.0 (55.1%)
Male	1320.0 (43.6%)	401.0 (49.9%)	1721.0 (44.9%)
Eye colour			
Blue	2181.0 (76.1%)	623.0 (81.5%)	2804.0 (77.2%)
Green	120.0 (4.2%)	33.0 (4.3%)	153.0 (4.2%)
Hazel	494.0 (17.2%)	99.0 (13.0%)	593.0 (16.3%)
Brown	71.0 (2.5%)	9.0 (1.2%)	80.0 (2.2%)
Family history of skin cancer (including melanoma)			
No	2749.0 (90.8%)	655.0 (81.6%)	3404.0 (88.9%)
Yes	277.0 (9.2%)	148.0 (18.4%)	425.0 (11.1%)
Family history of melanoma			
No	2898.0 (95.8%)	740.0 (92.2%)	3638.0 (95.0%)
Yes	128.0 (4.2%)	63.0 (7.8%)	191.0 (5.0%)
Born in Australia	2279.0 (75.3%)	654.0 (81.4%)	2933.0 (76.6%)
Currently smoke tobacco	234.0 (14.8%)	65.0 (17.1%)	299.0 (15.2%)
Ever smoked tobacco	1585.0 (52.4%)	380.0 (47.3%)	1965.0 (51.3%)
Current pterygium			
No	2595.0 (89.4%)	661.0 (85.3%)	3256.0 (88.5%)
Yes	309.0 (10.6%)	114.0 (14.7%)	423.0 (11.5%)
Previous pterygium excision			
No	2953.0 (97.6%)	768.0 (95.6%)	3721.0 (97.2%)
Yes	73.0 (2.4%)	35.0 (4.4%)	108.0 (2.8%)
Current pterygium or previous excision			
No	2551.0 (87.8%)	641.0 (82.6%)	3192.0 (86.7%)
Yes	354.0 (12.2%)	135.0 (17.4%)	489.0 (13.3%)
Myopia (≤ −0.50 D)			
No	1618.0 (82.2%)	421.0 (84.2%)	2039.0 (82.6%)
Yes	351.0 (17.8%)	79.0 (15.8%)	430.0 (17.4%)

*Note: n* (%) are shown for categorical variables and mean (SD) for continuous variables.

### 3.2 Agreement Between Self‐Reported and Administratively Recorded Diagnoses

There was slight agreement between self‐reported data and administrative records for both keratinocyte cancer (*κ* = 0.24) and cutaneous melanoma diagnoses (*κ* = 0.37; Table 2). Eighty people self‐reported a cutaneous melanoma diagnosis but had no corresponding administrative record. Of these, 69 (86%) had an administrative record of a keratinocyte cancer. Approximately one‐third (38%) of participants with an administrative record of cutaneous melanoma did not self‐report a melanoma diagnosis; of these, only one self‐reported a non‐specific skin cancer diagnosis.

**TABLE 2:** Agreement between self‐reported and linked keratinocyte and melanoma skin cancer history.

	Linked: No	Linked: Yes	Kappa (95% CI)
Melanoma			0.37 (0.27, 0.47)
Self‐reported: No	3688	18	
Self‐reported: Yes	80	30	
Keratinocyte			0.24 (0.19, 0.28)
Self‐reported: No	3045	473	
Self‐reported: Yes	154	145	

*Note:* Data shown are number of participants. CI: confidence interval. Excludes participants who self‐reported melanoma diagnosis prior to 1982 (*n* = 11) or keratinocyte cancer diagnosis prior to 1980 (*n* = 7).

### 3.3 Logistic Regression

On univariable regression, only pterygium was associated with keratinocyte cancer (current pterygium: OR = 1.45, 95% confidence interval [CI]: 1.15, 1.83; pterygium excision: OR = 1.87, 95% CI: 1.24, 2.82). Pterygium was not associated with melanoma on univariable regression (OR = 1.44, 95% CI: 0.70, 2.97) and nor was myopia or CUVAF area associated with melanoma (myopia: OR = 1.09, 95% CI: 0.45, 2.63; CUVAF, per mm^2^ 0.99, 95% CI: 0.95, 1.04) or keratinocyte cancer (myopia: OR = 0.88, 95% CI: 0.68, 1.15; CUVAF, per mm^2^ 1.01, 95% CI: 0.99, 1.02). After adjusting for covariates, both a current pterygium or a self‐reported previous pterygium excision were associated with a keratinocyte cancer diagnosis (Table 3).

**TABLE 3:** Results of multivariable logistic regression analysis of association between cutaneous melanoma or keratinocyte cancer against primary exposure variables.

	Melanoma				Keratinocyte			
	Melanoma/no melanoma^a^	Odds ratio	95% CI	*p*	Keratinocyte/no keratinocyte^a^	Odds ratio	95% CI	*p*
Current pterygium								
No	47/3209	Referent	—	—	654/2602	Referent	—	—
Yes	7/414	1.16	0.55, 2.42	0.70	113/310	1.33	1.05, 1.69	0.019
Pterygium excision								
No	57/3664	Referent	—	—	760/2961	Referent	—	—
Yes	2/106	0.90	0.21, 3.87	0.89	35/73	1.85	1.21, 2.82	0.005
CUVAF area (per +1 mm^2^)		0.98	0.93, 1.03	0.38		1.00	0.99, 1.02	0.54
Myopia								
No	28/2011	Referent	—	—	417/1622	Referent	—	—
Yes	6/424	1.24	0.51, 3.03	0.63	79/351	0.96	0.74, 1.25	0.77

*Note:* All logistic regression models are adjusted for age, sex, eye colour, born in Australia, smoking history and self‐reported family history of either melanoma—for melanoma outcome—or skin cancer—for keratinocyte cancer outcome.

^a^ Numbers are based on observed data and do not reflect the use of multiple imputation used to derive the odds ratio, 95% confidence intervals and *p* values.

### 3.4 Sensitivity Analyses

Results were largely unchanged (Table 4), with a substantial change to the effect estimate only seen between myopia and melanoma; however, there was a large amount of uncertainty around this estimate.

**TABLE 4:** Sensitivity analyses showing results of multivariable logistics regression analyses when limiting to only participants born in Australia (*n* = 2933).

	Melanoma				Keratinocyte			
	Melanoma/no melanoma^a^	Odds ratio	95% CI	*p*	Keratinocyte/no keratinocyte^a^	Odds ratio	95% CI	*p*
Current pterygium								
No	39/2419	Referent	—	—	528/1930	Referent	—	—
Yes	8/360	1.16	0.53, 2.54	0.71	96/272	1.21	0.93, 1.57	0.15
Pterygium excision								
No	46/2794	Referent	—	—	614/2226	Referent	—	—
Yes	2/91	0.96	0.22, 4.178	0.96	32/61	1.89	1.20, 2.97	0.006
CUVAF area (mm^2^)		0.98	0.93, 1.03	0.40		1.01	0.99, 1.02	0.32
Myopia								
No	25/1534	Referent	—	—	339/1220	Referent	—	—
Yes	3/315	0.88	0.29, 2.68	0.82	60/258	0.94	0.69, 1.29	0.70

*Note:* Pterygium and myopia odds ratios are the odds of melanoma or keratinocyte cancer if in the ‘yes’ group compared to the ‘no’ group. Odds ratio for CUVAF area is change in odds of melanoma or keratinocyte cancer with a 1 mm^2^ increase in mean CUVAF area. All results are from multiple logistic regression models adjusted for age, sex, eye colour, being born in Australia, smoking history and self‐reported family history of either melanoma (for the melanoma outcome) or skin cancer (for the keratinocyte cancer outcome).

^a^ Numbers are based on observed data and do not reflect the use of multiple imputation used to derive the odds ratio, 95% confidence intervals and *p* values.

## 4 Discussion

We identified a positive association between pterygium, either current or previously excised, and higher odds of keratinocyte cancer. Despite being highly correlated with pterygium prevalence [16, 27], CUVAF area was not associated with either melanoma or keratinocyte skin cancer history nor was myopia.

To capture as many skin cancer diagnoses as possible, we combined self‐reported and administratively captured diagnoses. A Swedish study found that self‐report had a sensitivity of 52% and 70% for detecting administratively recorded keratinocyte and melanoma skin cancer, respectively [28]. A more recent study also set in regional Australia [29] found similar results to the current study regarding melanoma reporting: agreement with administrative data was poor (kappa = 0.27) and approximately 80% of participants who self‐reported a melanoma diagnosis did not have a corresponding administrative record. This highlights the limitations of self‐reported skin cancer diagnosis. By combining self‐reported and linked data, we aimed to minimise misclassification caused by under‐reporting and incorrect reporting of skin cancer diagnoses. Our decision to categorise participants who self‐reported a melanoma diagnosis but had no corresponding administrative record as having a keratinocyte cancer diagnosis was supported by the high prevalence of administrative keratinocyte diagnoses in this group (86%). Nevertheless, there remains potential for misclassification of skin cancer in the current study, which would bias the findings of associations towards the null.

Two previous studies using linked health data have found an association between pterygium excision and increased skin cancer risk. A WA study [30] reported that pterygium excision was associated with a 24% higher risk of subsequent cutaneous melanoma diagnosis. A Taiwanese study [31] using national health insurance data found that pterygium was associated with approximately 60% higher risk of subsequent non‐melanoma skin cancer excision. However, this study did not find an association between pterygium excision and cutaneous malignant melanoma risk, possibly because of the small number of melanoma cases (*n* = 23). In our study, the odds ratios and 95% confidence intervals were consistent with a small to medium association between a keratinocyte cancer diagnosis and a current pterygium (OR = 1.33) or a previous pterygium excision (OR = 1.85). This suggests having a pterygium excised may be more strongly associated with a keratinocyte cancer diagnosis; however, as pterygium excision was self‐reported, caution in comparing these associations is warranted [32]. Taken in context, our results support the conclusion that individuals who have had a pterygium or pterygium excised have a higher likelihood of a keratinocyte skin cancer diagnosis, and vice versa, and they may also have a higher likelihood of a cutaneous melanoma diagnosis, and vice versa.

CUVAF area is strongly associated with greater amounts of past sun exposure [13, 16, 27] but there are limited reports on its association with skin cancer. Although CUVAF area and pterygium are strongly correlated [16, 27], CUVAF area itself was not associated with skin cancer history. A sub‐group analysis of the first 1861 participants of the BHAS [32] found approximately twice the prevalence of myopia in those who didn't self‐report a skin cancer (21.6%) vs. those with a skin cancer (11.9%). We were unable to replicate this finding, possibly because we included both Phase 1 and 2 data and therefore captured participants with an older age at skin cancer diagnosis or because the inclusion of administratively recorded diagnoses in the current analysis also substantially increased the prevalence of captured cases of skin cancer (21.0% vs. 7.7%) [32].

While past UVR exposure is a well‐established risk factor for skin cancer, the lack of association between skin cancer and CUVAF or myopia could be explained by: (i) insufficient power to detect a true association, particularly with respect to melanoma, (ii) measurement limitations, particularly for myopia where substantial data were missing, (iii) residual confounding, including an alternative, shared exposure that links skin cancer and pterygium but not skin cancer and myopia or CUVAF area or (iv) differences in the relevant timing of UV exposure that leads to skin cancer, pterygium, CUVAF or myopia.

An alternative, shared exposure linking skin cancer with pterygium but not myopia or CUVAF may be failure of DNA repair mechanisms after a UVR‐mediated insult, which is a common step in the development of both pterygium and skin cancers [33]. This link is particularly prominent in patients with xeroderma pigmentosum, a rare autosomal recessive disorder arising from genetic variants impairing nucleotide excision repair, who are prone to the development of both skin cancers (10 000‐fold increased risk [34]) and pterygia (31% incidence in one cohort in Maryland, USA [35]). Genetic variants in the *XRCC1* [36] and *hOGG1* [37]—genes related to DNA repair—have also been associated with altered pterygia risk. Further, increased methylation, which leads to reduced expression of genes related to DNA repair, have been reported in pterygium compared to control conjunctival tissue [38]. Thus, it may be that patients who develop both skin cancer and pterygium have not only been exposed to a UVR‐mediated cellular insult, but may be more likely to have mildly deficient DNA repair mechanisms, explaining the shared risk between keratinocyte cancer and pterygium, but not with CUVAF.

An alternative explanation for the lack of association with CUVAF and myopia relates to the age and pattern at UVR exposure relevant to skin cancer development. Cutaneous melanoma risk is most strongly linked to intermittent, high‐intensity UVR (e.g., causing sunburns) early in life, prior to age 25 years [25, 39]. BCC is similarly associated with intermittent patterns of UVR exposure and sunburns in childhood while SCC is more strongly associated with high cumulative lifetime UVR exposure [39, 40]. Thus, CUVAF area, which we expect to be marker of medium‐term UVR exposure, may not represent an appropriate exposure period for BCC or cutaneous melanoma, while any association with SCC may have been diluted by pooling keratinocyte cancers. Myopia is related to low time outdoors in childhood and adolescence [13] and thus would be expected to correlate most strongly with cutaneous melanoma or BCC. However, the small number of melanoma cases limited our ability to precisely estimate this association. Further, use of skin protection without ocular sun protection and vice versa could cause a disconnect between ocular markers of sun exposure and skin cancers, biasing towards the null.

### 4.1 Strengths and Limitations

Strengths to the study include the large sample size from a single location (latitude 33.7° S), the inclusion of linked health data for outcome assessment, and the objective assessment of CUVAF area, pterygium and myopia. Limitations include the small number of participants with melanoma, which greatly reduced our ability to estimate associations with precision, the reliance on self‐reported data to identify all cases of keratinocyte cancer, the inability to distinguish between BCC and SCC among keratinocyte cancers or between melanoma in situ and carcinoma in situ in ICD‐9 codes, and the use of self‐reported data to assess past pterygium excision and skin cancer family history. The exposures used were all proxy measures for past UVR exposure and are not expected to be causally related to skin cancer. Further, myopia data were missing for 30% of participants due to device malfunction, which we attempted to mitigate through use of multiple imputation. As this is a cross‐sectional analysis, we are unable to infer the direction of association or causation. The study results are not generalisable to non‐European ancestry and higher skin pigmentation groups, who are typically at lower risk of skin cancers.

### 4.2 Conclusions

A current pterygium, or a self‐reported history of pterygium excision, is associated with a diagnosis of keratinocyte cancer and, possibly, also cutaneous melanoma. While it is unclear if pterygia precede skin cancer onset, people with a pterygium should be advised of their higher risk of skin cancer and need for increased self‐monitoring or professional monitoring of naevi and skin lesions.

## Funding

Core funding of the Busselton Healthy Ageing Study is provided by the Federal Government of Australia, the Government of Western Australia (Department of Health and Department of Jobs, Tourism, Science and Innovation), the National Health and Medical Research Council (NHMRC) Equipment Grant scheme, the City of Busselton and private donations to the Busselton Population Medical Research Institute. Samantha Lee is funded by a Western Australia Future Health and Research Innovation Emerging Leaders Fellowship. David Whiteman is supported by an Investigator Grant (2026567) from the National Health and Medical Research Council of Australia (NHMRC). David Mackey is supported by a Stan Perron Charitable Foundation health research grant.

## Conflicts of Interest

The authors declare no conflicts of interest.

## Data Availability

The data that support the findings of this study are available from Busselton Population Medical Research Institute. Restrictions apply to the availability of these data, which were used under license for this study. Data are available from the author(s) with the permission of Busselton Population Medical Research Institute at https://bpmri.org.au/research/database‐access.html.
